# Management of Brachial Artery Pseudoaneurysms in Intravenous Drug Abusers

**DOI:** 10.7759/cureus.12315

**Published:** 2020-12-27

**Authors:** Khalil Ur Rehman, Muhammad Fahad Berlas, Najam U Din, Ghulam Ali, Farhina Salahuddin, Asma Mumtaz

**Affiliations:** 1 Department of Vascular Surgery, Shaheed Mohtarma Benazir Bhutto Institute of Trauma, Karachi, PAK

**Keywords:** ligation, brachial artery, pseudoaneurysm, vascular surgery, excision

## Abstract

Objective

To determine the outcomes of the ligation and excision of brachial artery pseudoaneurysm in IV drug abusers without revascularization.

Methodology

This retrospective observational study was conducted at the vascular surgery department Shaheed Muhtarma Benazir Bhutto trauma center Karachi from January 2019 to June 2020. All the patients with a history of intravenous drug abuse presented with pulsatile mass at or near cubital fossa, diagnosed as pseudoaneurysm, age ranging from 18-70 years, and of either gender were included in the study. Patients with pseudo-aneurysm secondary to trauma, hemodialysis, arteriovenous fistula, location other than cubital fossa, and whom primary revascularization was performed were excluded. The recorded data entered and analyzed using SPSS 20.0 (IBM Corp., Armonk, NY).

Results

A total of 20 intravenous drug addicts were included in the study. The mean age was of 31.10 ± 7.80 years, and the mean duration of addiction was 2.24 ± 1.16 years. The right arm is affected in almost two-thirds of patients. The most common presentation in the emergency department was ruptured pseudo-aneurysm with bleeding (65%), followed by oozing with pulsatile mass (30%), and infected pulsatile mass (5%). The outcome was Limb salvage (100%), and none of the patients had developed threatened ischemia of the arm or required amputation.

Conclusion

The ligation and excision of the pseudo-aneurysm, without revascularization, is a safe and effective treatment option for the management of pseudoaneurysm of the brachial artery secondary to intravenous drug addiction.

## Introduction

The use of illicit drugs in society is at a surge, and its impact on our youth and skilled people is problematic. A detailed report by the United Nations Office on Drugs and Crime (UNODC) revealed an estimate of 35 million drug-affected people globally [1]. Pakistan is one of the most drug-affected countries in the world-affecting, about 7.8 million[2]. Intravenous drug addiction (IVDA) is an extremely challenging social and health issue. Early death among intravenous drug addicts has an association with overdosage, suicide, trauma, and infections like HIV (human immunodeficiency virus) and Hep-C (hepatitis C) [3] as well as various vascular complications [4].

Arterial Pseudoaneurysm (False Aneurysm) differs from true aneurysms. They lack all three normal elements of the arterial wall, although a rare complication (a potential limb and life-threatening). It is formed due to repetitive injury to the vessel wall leading to a communicating hematoma in the surrounding tissue [5,6]. Clinically diagnosed as an expanding, pulsatile, painful mass often accompanied by erythema, induration with a history of intravenous drug addiction. The examination may reveal decreased temperature, thrill or an audible bruit, cyanosis, loss of pulsation, and distal paresthesia due to nerve compression. A pseudo-aneurysm can distinguish from a true aneurysm by lacking all three vessel walls and from waveform in duplex Doppler ultrasound[7,8]^.^

The femoral artery pseudo-aneurysm is the most common vascular complication of IVDA; however, the brachial artery's involvement is not uncommon [9]. Brachial artery pseudo-aneurysm presented commonly as an expanding, pulsatile, infected, painful mass, with/ without peripheral neurovascular compromise, especially in dermatomes of the median nerve [6]. The traditional treatment option for this complex problem is ligation and excision of pseudo-aneurysm and debridement of surrounding tissue if infected [9]. This surgical treatment has more favourable outcomes than those performed elsewhere due to good collaterals eliminating the need for reconstruction[10].

This research aims to evaluate the outcomes of ligation and excision of brachial artery pseudo-aneurysm of Intravenous drug addicts with radical debridement (if infective) without primary revascularization in our hospital. Currently, there is no national data available for specifically brachial artery pseudo-aneurysm. This research would be a valuable input for the management of this complicated vascular emergency condition.

## Materials and methods

This retrospective observational study was conducted at the Department of Vascular Surgery at Shaheed Mohtarma Benazir Bhutto Institute (SMBBIT) Karachi after the Institutional Review Board's research proposal. The data collection included all intravenous drug abusers with brachial artery pseudo-aneurysm presented in our emergency department from January 2019 to June 2020. All patients who were intravenous drug abusers presented with pulsatile mass at or near cubital fossa, age ranging from 18-70 years, and of either gender were included in the study. Patients with pseudo-aneurysm secondary to trauma, hemodialysis, arterio-venous fistula, location other than cubital fossa, and whom primary revascularization was performed were excluded. Patients demographic data, side, mode of presentation, duration of addiction, and outcome variables (limb salvage, threatened limb ischemia, amputation, and mortality) were recorded in proforma during the index admission.

Procedure

**Figure 1:**
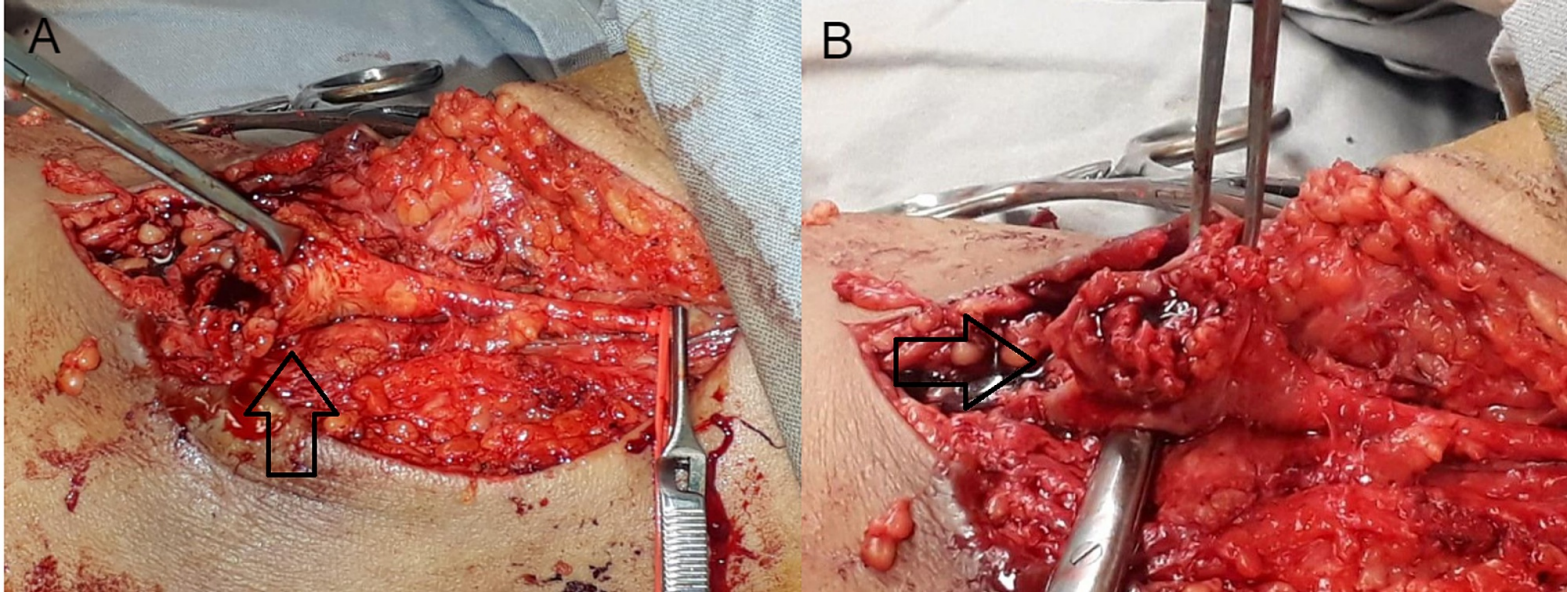
Intra-operative exposure of brachial artery pseudoaneurysm. (A) Proximal control with the application of silastic loops and bulldog clamp. (B) Complete dissection of pseudoaneurysm from surrounding tissues.

Under general anaesthesia, the patient was placed in the supine position with the involved upper limb in abducted position. A curvilinear incision over the cubital fossa was given. Hemostasis achieved by proximal and distal control with subsequent suture ligation, followed by excision of pseudo-aneurysm, and debridement of the surrounding tissue, leaving the wound to heal by secondary intention (Figure 1A, B).

The patients remained under observation for over 72 hours. All outcomes were determined in the same admission as it is common to present threatened ischemic limb in 72 hours, requiring revascularization procedure or amputation.

The recorded data entered and analyzed using SPSS 20.0 (IBM Corp., Armonk, NY). The quantitative data were analyzed as mean ± SD while qualitative data as frequency and percentages. Data were stratified for age, gender, duration of addiction, side of the arm, mode of presentation, hand dominance, and outcome variables.

## Results

This study included a total of 20 patients who were presented in our emergency department with a brachial artery pseudo-aneurysm and with a positive history of intravenous drug addiction. Out of these 20 patients, there were 14 males (70%) and 6 females (30%) with a mean age of 31.10 ± 7.80 years (Table 1).

**Table 1:** Descriptive statistical analysis.

Variables (n = 20)		Mean±SD/frequency
Age (years) (18-70years)		31.10 ± 7.80
Gender	Male	14 (70%)
	Female	6 (30%)
Mode of Admission	Emergency	20 (100%)
	OPD	0
Side of injury	Right	12 (60%)
	Left	8 (40%)
Co-morbid	HBs Ag+ve	10 (50%)
	AntiHCV +ve	4 (20%)
	HIV +ve + HBV/HCV +ve	6 (30%)
Presentation	Ruptured pseudoaneurysm with bleeding	13 (65%)
	oozing with a pulsatile mass	6 (30%)
	infected pulsatile mass	1 (5%)
	Misdiagnosed and underwent I& D	0
Duration of addiction (years)		2.24 ± 1.16
Outcome	Limb Salvage	20 (100%)
	Threatened Limb Ischemia	0
	Amputation	0
	Mortality	0

**Figure 2:**
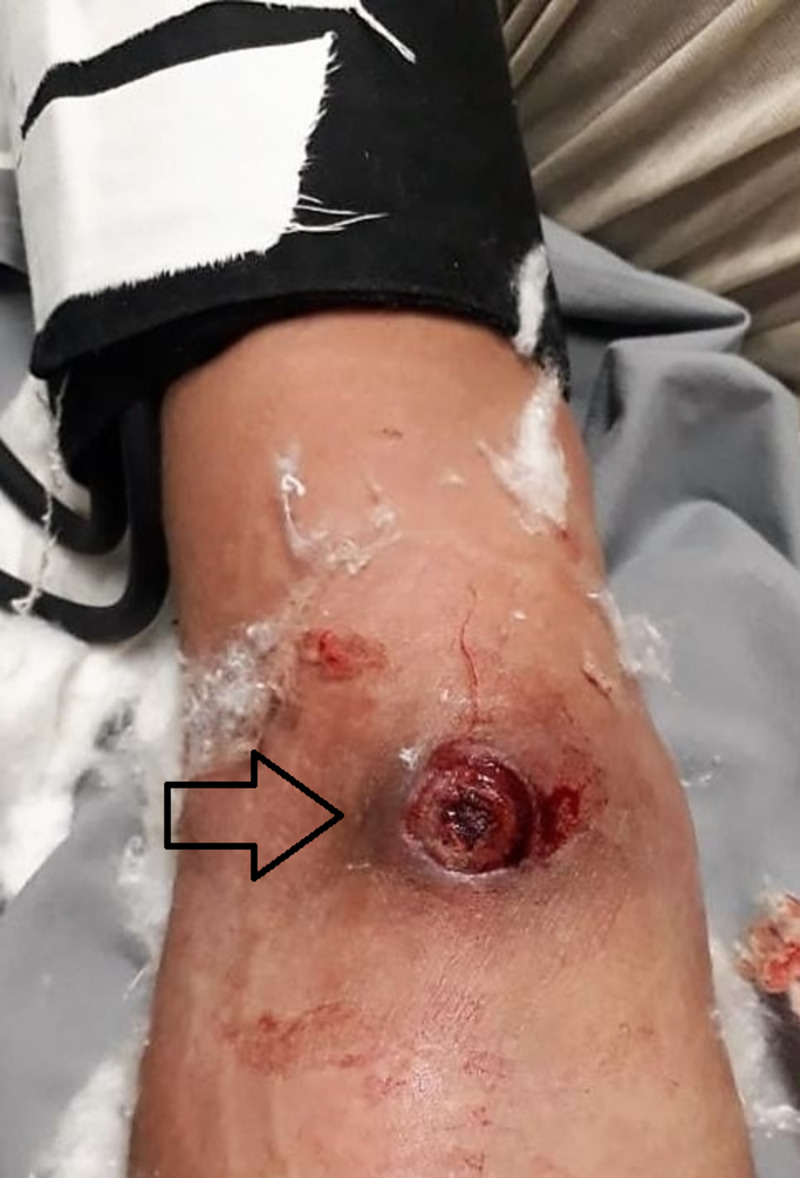
Patient with ruptured brachial artery pseudoaneurysm. The figure shows skin erythema around a ruptured brachial artery pseudoaneurysm with the proximal application of BP cuff to manage profuse bleeding at presentation.

The mean duration of IV addiction to substance abuse was 2.24 ± 1.16 years, and the right arm was affected in 60% of cases whereas the left was affected in 40% of cases. The most common presentation in the emergency department was ruptured pseudo-aneurysm with bleeding (65%), followed by oozing with pulsatile mass (30%), and infected pulsatile mass (5%) (Figure 2). All patients presented in our institute's emergency department (100%) are associated with infective diseases. 

All patients underwent ligation and excision of pseudo-aneurysm with debridement of surrounding infective tissue in one patient, leaving the wound to heal by secondary intention and without primary revascularization procedure. None of the patients has developed threatened ischemia of the arm or required amputation. 

## Discussion

The most common causes of pseudo-aneurysm are diagnostic or therapeutic vascular interventions, intravenous drug addiction, and trauma [11]. Pseudo-aneurysms following drug injections are frequently documented in the literature, however, mostly focusing on the femoral artery pseudo-aneurysm treated surgically with ligation, debridement (if infected), primary or secondary revascularization, thrombin injection (ultrasound-guided), and arterial puncture closure [12-14]. Though the cubital fossa is the preferred injection site for most illicit drug users, post-injection arterial complications in the brachial artery are rarely reported [15]. In parental drug abusers, repeated injections under non-sterile conditions through the arterial wall and sharing of needles favour the transmission of vertically transmitted infections like hepatitis B, C, and HIV and the development of the pseudo-aneurysm [16]. This study noted an upsurge in the frequency of brachial artery pseudo-aneurysm compared to other studies where femoral vessel involvement is quite common. Besides increased frequency, all patients were associated with other infective diseases such as Hep B, affecting about two-thirds of patients, followed by Hepatitis C and HIV, as shown in Table 1.

A study in Pakistan shows male preponderance (93.5%) in drug addiction presented in the emergency department with peripheral arterial complications [17]. In contrast, in our study, we found one-third of drug-addicted patients were female (30%). Our study's most common presentation is bleeding from ruptured pseudo-aneurysm (about 65%), compared to another study, where 70% with bleeding from a ruptured pseudo-aneurysm, however, that study included only two patients of brachial artery pseudoaneurysm [10].

In intravenous drug addicts, repeated needling leads to fibrosis of veins, and tissue damage to the surrounding area makes it more challenging for treatment. Various interventions for the management of pseudo-aneurysm have been documented in the literature with no clear consensus of prioritizing the management. Treatment options for the brachial artery pseudoaneurysm are simple ligation and excision, primary or secondary revascularization, endovascular stenting, percutaneous thrombin injection, and ultrasound-guided compression [9,18]. In this study, all patients presented late in the emergency department with bleeding from ruptured pseudo-aneurysm or oozing from pulsatile mass, and underwent simple ligation and excision pseudo-aneurysm (with debridement of surrounding tissue if infected) without primary revascularization. There has been no threatened ischemia requiring revascularization procedure or amputation. With early intervention, no mortality has been seen, which may be due to rich collateral blood supply in the upper limb.

This study provides promising results of ligation and excision of the brachial artery pseudo-aneurysm in intravenous drug addicts. However, it cannot be generalized as long-term follow-up is required to foresee and predict this procedure's outcome. Hence, this is the limitation of this study.

## Conclusions

In conclusion, the ligation and excision of the pseudo-aneurysm, without revascularization, is an acceptable, safe, and effective treatment option for managing the pseudo-aneurysm of the brachial artery secondary to IVDA. However, further studies are required, covering a more extended period and follow-up to formulate and strategize the guideline to treat this devastating vascular complication in intravenous drug addicts.
